# Diabetes and Perinatal Mortality in Twin Pregnancies

**DOI:** 10.1371/journal.pone.0075354

**Published:** 2013-09-18

**Authors:** Zhong-Cheng Luo, Yan-Jun Zhao, Fengxiu Ouyang, Zu-Jing Yang, Yu-Na Guo, Jun Zhang

**Affiliations:** 1 Ministry of Education, Shanghai Key Laboratory of Children’s Environmental Health, Xinhua Hospital, Shanghai Jiao-Tong University School of Medicine, Shanghai, China; 2 Department of Obstetrics and Gynecology, Xinhua Hospital, Shanghai Jiao-Tong University School of Medicine, Shanghai, China; 3 Department of Obstetrics and Gynecology, International Peace Maternity and Child Health Hospital, Shanghai Jiao-Tong University School of Medicine, Shanghai, China; 4 Department of Obstetrics and Gynecology, Sainte-Justine Hospital, University of Montréal, Montreal, Quebec, Canada; University of Tennessee Health Science Center, United States of America

## Abstract

**Background:**

Diabetes in pregnancy has been associated with a paradoxically reduced risk of neonatal death in twin pregnancies. Risk “shift” may be a concern in that the reduction in neonatal deaths may be due to an increase in fetal deaths (stillbirths). This study aimed to clarify the impact of diabetes on the risk of perinatal death (neonatal death plus stillbirth) in twin pregnancies.

**Methods:**

This was a retrospective cohort study of twin births using the largest available dataset on twin births (the U.S. matched multiple birth data 1995-2000; 19,676 neonates from diabetic pregnancies, 541,481 from non-diabetic pregnancies). Cox proportional hazard models were applied to estimate the adjusted hazard ratios (aHR) of perinatal death accounting for twin cluster-level dependence.

**Results:**

Comparing diabetic versus non-diabetic twin pregnancies, overall perinatal mortality rate was counterintuitively lower [2.1% versus 3.3%, aHR 0.70 (95% confidence intervals 0.63-0.78)]. Individually, both stillbirth and neonatal mortality rates were lower in diabetic pregnancies, but we identified significant differences by gestational age and birth weight. Diabetes was associated with a survival benefit in pregnancies completed before 32 weeks [aHR 0.55 (0.48-0.63)] or with birth weight <1500 g [aHR 0.61 (0.53-0.69)]. In contrast, diabetes was associated with an elevated risk of perinatal death in pregnancies delivered between 32 and 36 weeks [aHR 1.38 (1.10-1.72)] or with birth weight >=2500 g [aHR 2.20 (1.55-3.13)].

**Conclusions:**

Diabetes in pregnancy appears to be “protective” against perinatal death in twin pregnancies ending in very preterm or very low birth weight births. Prospective studies are required to clarify whether these patterns of risk are real, or they are artifacts of unmeasured confounders. Additional data correlating these outcomes with the types of diabetes in pregnancy are also needed to distinguish the effects of pre-gestational vs. gestational diabetes.

## Introduction

Diabetes mellitus affects 2 to 10% of pregnancies, and most (about 90%) of diabetes in pregnancy are gestational diabetes [1-3]. Pregnancy is a relatively “diabetogenic” state due to the increase in hormonal antagonists of the insulin (e.g. human placental lactogen) resulting in reduced tissue sensitivity to insulin [4]. The levels of such pregnancy-related diabetogenic hormones are higher in multiple versus singleton pregnancies due to the increased placental mass [5]. Hence, multiple pregnancies are more likely affected by gestational diabetes [6,7]. In general, diabetes in pregnancy has been associated with a number of adverse outcomes including congenital anomalies, preterm birth, macrosomia, neonatal hypoglycemia, and neonatal death [8-14]. However, virtually all previous studies on the impact of diabetes on perinatal outcomes did not address the differences between multiple versus singleton pregnancies. A recent study suggests a potential differential impact of diabetes on perinatal outcomes in multiple versus singleton pregnancies; there appears to be a “protective” effect of diabetes against neonatal death in twin (but not singleton) pregnancies [15]. A logical concern in data interpretation is that the reduction in neonatal deaths in twin diabetic pregnancies may be due to an increase in fetal deaths (risk shift). This study sought to clarify this effect by evaluating the risk of perinatal death (fetal death plus neonatal death) in twin diabetic pregnancies.

## Methods

This was a retrospective cohort-based analysis of twin births, using the U.S. National Center for Heath Statistics (NCHS)’s *matched multiple birth data 1995-2000* (the largest available linked multiple birth dataset) [16]. The NCHS matched multiple birth data contain information on maternal and pregnancy characteristics and perinatal and infant mortality for all multiple births in years 1995-2000 in the United States of America. We restricted the analyses to twins because the risks of perinatal death for triplets and high-order births are too high and less likely related to diabetes in pregnancy. As the study was aimed to evaluate the impact of diabetes on perinatal death, we excluded births with missing information on diabetes in pregnancy. Most states reported/registered fetal deaths at 20 or more completed weeks of gestation, but a few states reported fetal deaths below 20 weeks (with uncertain completeness). To reduce uncertainties in data quality in such large population-based vital registry data, we excluded births recorded at extreme gestational ages (<20 or >42 weeks) or extreme birth weights (<200 g, or >6000 g), or implausible birth weight for gestational age [17]. We did not exclude births with congenital anomalies because diabetes may increase the risk of perinatal death through increasing the risk of birth defects (on the causal pathway). The final study cohort included 281,505 twin pregnancies (561,157 twin births). The number of twin births was less than the double of twin pregnancies because there were missing and ineligible records in some twin sets. Research ethics approval was waived by the Shanghai Xinhua hospital research ethics board because the study was based on the anonymized matched multiple birth database downloadable from the NCHS website.

There was no information on the clinical subtypes of diabetes in pregnancy in the NCHS birth data. During the study reference period, universal screen for gestational diabetes was in place. The 3-hour 100 g oral glucose tolerance test (OGTT) was routinely used for the diagnosis of gestational diabetes, according to the diagnostic criteria recommended by the American Diabetes Association (ADA) or the National Diabetes Data Group [1,3]. There was no information on the diagnosis criteria (ADA or NDDG?) in the NCHS birth data,

Primary outcome was perinatal death. Secondary outcomes included the components of perinatal death - stillbirth (fetal deaths ≥20 weeks gestation) and neonatal death [deaths during the first four weeks (0-27 days) of postnatal life]. The primary exposure was diabetes in pregnancy. The study co-variables included maternal race (white, black, others), age (<20, 20-34, 35+ y), education [<12, 12 (high school graduation), 13-15 (some post-secondary), and 16+ y (college or higher)], marital status (not married; married), parity (primiparous, multiparous), maternal smoking (yes, no), the presence of other reported major maternal illnesses including chronic hypertension, heart disease, acute or chronic lung disease, genital herpes, renal disease and Rh sentization (yes, no), fetal sex (boy, girl), mode of delivery (caesarean, vaginal), gestational age (weeks) and birth weight (grams).

Cox proportional hazard models for clustered data [18] were applied to estimate the crude and adjusted hazard ratios (aHR) and 95% confidence intervals (CI) of perinatal death, stillbirth and neonatal death comparing diabetic vs. non-diabetic pregnancies, using a robust sandwich covariance estimate to account for the twin-cluster level dependence. The unit of analysis was the twin pregnancy (cluster) for the exposure (diabetes), but the fetus for the outcomes (perinatal death, stillbirth, neonatal death). The time variable was the duration of gestation. The aHRs were controlled for maternal race, age, marital status, parity, smoking, the presence of other reported maternal illnesses, fetal sex and mode of delivery. To gain insight into clinical characteristics of risk changes, the HRs of perinatal death were examined by important clinical characteristics: mode of delivery (caesarean, vaginal), gestational age (very preterm <31 weeks, mild preterm 32-36 weeks, full-term ≥37 weeks), birth weight (very low <1500 g, low 1500-2499 g, normal ≥2500 g), and fetal growth – birth weight small, appropriate or large for gestational age (SGA <10^th^ percentile, AGA 10-90^th^ percentiles, LGA >90^th^ percentile, according to sex- and gestational age-specific birth weight percentiles for non-malformation births to mothers without smoking and without any reported major maternal illness in the study cohort). For gestational age group-specific mortality rates and HRs, fetuses-at-risk approach was applied to avoid a potential “collider” effect of stratification by gestational age at birth [19]. The fetuses-at-risk denominator is the number of all fetuses at risk of death (both born and yet unborn babies).

Births with missing data on co-variable (for most variables, missing <5%) were allowed to drop out in all multivariate adjustment models, except for smoking and mode of delivery for which the proportion of missing was high (17.7% for smoking, 36.6% for mode of delivery); the missing was included as a valid category to avoid large numbers of drop-outs in the adjusted risk models. All data management and analyses were carried out using Statistical Analysis System, version 9.2 (SAS Institute, Cary, North Carolina). Two-tailed p values <0.05 were considered statistically significant.

When comparing risks in diabetic and non-diabetic pregnancies for perinatal death, stillbirth and neonatal death, ad hoc calculations indicated a power of >99% to detect a risk increase or decrease of 30% or greater.

## Results

Diabetes was reported in 9855 of 281505 twin pregnancies (3.5%) in the study cohort. Significant differences were observed in maternal, pregnancy and newborn characteristics comparing twin diabetic versus non-diabetic pregnancies (Table **1**). In twin diabetic pregnancies, mothers were more likely to be black (6.9% vs. 3.8%), ≥35 years of age (28.8% vs. 18.5%), to have reported any other maternal major illness (10.6% vs. 4.6%), or to have a caesarean section delivery (60.8% vs. 51.9%). As expected, preterm (61.4% vs. 56.1%) or large-for-gestational-age (13.3% vs. 9.3%) births and congenital anomalies (2.8% vs. 2.1%) were more frequent comparing diabetic vs. non-diabetic twin pregnancies. Extremely preterm births were slightly less frequent (10.5% vs. 12.1%), while mild preterm preterm births more frequent (50.9% vs. 44.0%) in diabetic pregnancies. Low birth weight (52.5% vs. 54.6%) and small-for-gestational-age (9.9% vs. 10.8%) births were slightly less frequent in diabetic pregnancies. Among extreme preterm births comparing diabetic vs. non-diabetic pregnancies, SGA was slightly less frequent (10.0% vs. 11.7%), while LGA was similarly frequent (10.1% vs. 9.7%, p=0.48).

**Table 1:** Maternal, pregnancy and newborn characteristics in diabetic versus non-diabetic twin pregnancies in the study population, U.S. 1995-2000 [16].

	Diabetic	Non-diabetic	P
	pregnancy	pregnancy	
Number of twin pregnancies	9855	271650	
Number of included births	19676	541481	
*Mothers, n (%*)			
Race			<0.0001
Black	681 (6.9)	10348 (3.8)	
White	7808 (79.2)	214042 (78.8)	
Others	1366 (13.9)	48260 (17.4)	
Unmarried	1859 (18.9)	75265 (27.8)	<0.0001
Age (years)			<0.0001
<20	202 (2.1)	194244 (7.2)	
20-34	6820 (69.2)	204000 (74.4)	
>35	2833 (28.8)	50226 (18.5)	
Education			<0.0001
Lower than high school	1092 (11.2)	42665 (15.9)	
High School (12 y)	2873 (29.5)	82175 (30.6)	
Some college (13-15 y)	2255 (26.3)	61957 (23.1)	
College or higher (>16 y)	3209 (33.0)	81487 (30.4)	
*Pregnancies, n (%*)			
Primiparous	4076 (41.4)	113411 (41.8)	0.43
Maternal smoking ^ʂ^	721 (8.7)	23564 (10.6)	<0.0001
Other maternal major illness ^ʃ^	965 (10.6)	10870 (4.6)	<0.0001
Caesarean delivery ^ʂ^	3806 (60.8)	92785 (51.9)	<0.0001
*Newborns, n (%*)			
Preterm birth (<37 weeks)	12076 (61.4)	303881 (56.1)	<0.0001
Mild preterm (32-36 weeks)	10023 (50.9)	238499 (44.0)	<0.0001
Very preterm (<32 weeks)	2053 (10.4)	65382 (12.1)	<0.0001
Low birth weight (<2500 g)	10306 (52.5)	294903 (54.6)	<0.0001
SGA (<10^th^ percentile)*	1938 (9.9)	58284 (10.8)	<0.0001
LGA (>90^th^ percentile)*	2605 (13.3)	50141 (9.3)	<0.0001
Congenital anomalies	546 (2.8)	11448 (2.1)	<0.0001

Data presented are n (%). P values are from Chi-square tests for differences between diabetic and non-diabetic pregnancies.

* SGA=Small-for-gestational-age <10^th^ percentile, LGA=large-for-gestational-age >90^th^ percentile, according to birth weight percentiles in the study cohort.

ʃ Anyone or more of the following conditions: chronic hypertension, heart disease, acute or chronic lung disease, renal disease, genital herpes and RH sensitization.

ʂ There were significant numbers (>10%) of missing information on smoking (17.8% missing) and mode of delivery (36.6% missing). The percentages of smokers and caesarean section were based on births without missing information.

Overall, perinatal death occurred less frequently in twin diabetic vs. non-diabetic pregnancies [2.1% vs. 3.3%, aHR 0.70 (0.63-0.78)] (Table **2**). Stratified analyses revealed a significantly lower risk of perinatal death in twin diabetic pregnancies ending in very preterm [aHR 0.55 (0.48-0.63)], very low birth weight [aHR 0.61 (0.53-0.69)] or AGA [aRR 0.65 (0.57-0.75)] births. In contrast, diabetes was associated with a high risk of perinatal death in mild preterm [aHR 1.38 (1.10-1.72)] or normal birth weight [aHR 2.20 (1.55-3.13)] twins. The lower risk of perinatal death in diabetic twin pregnancies was observed in vaginal, but not caesarean section births.

**Table 2:** Perinatal mortality in diabetic versus non-diabetic twin pregnancies, U.S. matched multiple birth data 1995-2000 [16].

	**Perinatal**	**mortality (rate**)		
	Diabetic	Non-diabetic	Crude	Adjusted*
	Pregnancy	Pregnancy	HR (95% CI)	HR (95% CI)
	n/total (%)	n/total (%)		
All births (n=561157)	414/19676 (2.1)	17605/541481 (3.3)	**0.62 (0.56-0.69)**	**0.70 (0.63-0.78)**
Mode of delivery (^ʂ^ P=0.0005)				
Caesarean section	120/7577 (1.6)	3358/185528 (1.8)	0.88 (0.72-1.07)	0.94 (0.77-1.15)
Vaginal	95/4505 (2.1)	6480/158455 (4.1)	**0.52 (0.42-0.65)**	**0.58 (0.46-0.72)**
Unknown	199/7594 (2.6)	7767/197498 (3.9)	**0.60 (0.51-0.71)**	**0.65 (0.55-0.77)**
Gestational age ^ʃ^ (^ʂ^ P<0.0001)				
Very preterm (<32 weeks)	255/19676 (1.3)	14257/541481 (2.6)	**0.48 (0.41-0.55)**	**0.55 (0.48-0.63)**
Mild preterm (32-36 weeks)	114/17623 (0.65)	2249/476099 (0.47)	**1.36 (1.09-1.69)**	**1.38 (1.10-1.72)**
Term (>=37 weeks)	45/7600 (0.59)	1099/237600 (0.46)	1.31 (0.92-1.85)	1.31 (0.92-1.86)
Birth weight (^ʂ^ P<0.0001)				
Very low (<1500 g)	277/1774 (15.6)	14247/57983 (24.6)	**0.64 (0.57-0.71)**	**0.61 (0.53-0.69)**
Low (1500-2499 g)	72/8432 (0.84)	1730/236920 (0.73)	1.21 (0.92-1.59)	1.17 (0.89-1.55)
Normal (>=2500 g)	42/9326 (0.45)	590/244791 (0.24)	**2.21 (1.56-3.13)**	**2.20 (1.55-3.13)**
Fetal growth (^ʂ^ P<0.0001)				
SGA (<10^th^)	135/1938 (7.0)	5049/58284 (8.7)	**0.76 (0.62-0.92)**	0.82 (0.68-1.01)
AGA (10^th^-90^th^)	251/15133 (1.7)	12031/433056 (2.8)	**0.58 (0.51-0.67)**	**0.65 (0.57-0.75)**
LGA (>90^th^)	28/2605 (1.1)	525/50141 (1.1)	0.96 (0.62-1.49)	1.05 (0.68-1.63)

HR = Hazard ratio; CI = confidence interval

* Hazard ratios adjusted for maternal race, age, education, marital status, parity, smoking, other maternal major illnesses, fetal sex, mode of delivery and twin-cluster level dependence in Cox proportional hazard models.

ʃ Gestational age group-specific mortality rates and hazard ratios were calculated using the fetuses-at-risk denominator including all fetuses at risk of death (both born and unborn babies).

ʂ P value in test for interaction with diabetes in pregnancy in relation to the risk of perinatal mortality

Analyses on the components of perinatal death observed a mostly similar pattern for neonatal death and stillbirth (Tables **3** and **4**). Lower risks of both stillbirth and neonatal death were observed in twin diabetic pregnancies ending in very preterm or very low birth weight births. Higher risks of stillbirth [aHR 2.91 (1.73-4.87)] and neonatal death [aHR 1.81 (1.12-2.93)] were observed in twin diabetic pregnancies ending in normal birth weight births.

**Table 3:** Stillbirth in diabetic versus non-diabetic twin pregnancies, U.S. matched multiple birth data 1995-2000 [16].

	**Stillbirth**	**(rate**)		
	Diabetic	Non-diabetic	Crude	Adjusted*
	Pregnancy	Pregnancy	HR (95% CI)	HR (95% CI)
	n/total (%)	n/total (%)		
All births (n=561157)	168/19676 (0.85)	5807/541481 (1.07)	**0.78 (0.64-0.94)**	0.83 (0.69-1.01)
Mode of delivery (^ʂ^ P=0.0002)				
Caesarean section	33/7577 (0.44)	562/185528 (0.30)	**1.56 (1.03-2.35)**	**1.53 (1.01-2.32)**
Vaginal	33/4505 (0.73)	1499/158455 (0.95)	0.85 (0.57-1.27)	0.92 (0.62-1.37)
Unknown	102/7594 (1.34)	3746/197498 (1.9)	**0.63 (0.49-0.82)**	**0.68 (0.53-0.89)**
Gestational age ^ʃ^ (^ʂ^ P<0.0001)				
Very preterm (<32 wks)	77/19676 (0.39)	4035/541481 (0.75)	**0.51 (0.39-0.68)**	**0.57 (0.43-0.75)**
Mild preterm (32-36 wks)	66/17623 (0.37)	1180/476099 (0.25)	**1.52 (1.11-2.09)**	**1.53 (1.12-2.10)**
Term (>=37 wks)	25/7600 (0.33)	592/237600 (0.25)	1.30 (0.78-2.18)	1.28 (0.76-2.16)
Birth weight (^ʂ^ P<0.0001)				
Very low (<1500 g)	94/1774 (5.3)	3955/57983 (6.8)	**0.68 (0.53,0.87**)	**0.72 (0.56-0.93)**
Low (1500-2499 g)	33/8532 (0.39)	820/236920 (0.35)	1.25 (0.81-1.93)	1.19 (0.76, 1.84)
Normal (>=2500 g)	23/9326 (0.25)	258/244791 (0.11)	**2.90 (1.73-4.83)**	**2.91 (1.73-4.87)**
Fetal growth (^ʂ^ P<0.0001)				
SGA (<10^th^)	66/1938 (3.41)	2068/58284 (3.55)	0.90 (0.66-1.23)	0.95 (0.70-1.30)
AGA (10^th^-90^th^)	86/15133 (0.57)	3546/433056 (0.82)	**0.69 (0.53-0.90)**	**0.72 (0.55-0.95)**
LGA (>90^th^)	16/2605 (0.61)	193/50141 (0.38)	1.70 (0.92-3.15)	1.84 (0.99-3.41)

HR=Hazard ratio; CI= confidence interval

* Hazard ratios adjusted for maternal race, age, education, marital status, parity, smoking, other maternal major illnesses, fetal sex, mode of delivery and twin-cluster level dependence in Cox proportional hazard models.

ʃ Gestational age group-specific mortality rates and hazard ratios were calculated using the fetuses-at-risk denominator including all fetuses at risk of death (both born and unborn babies).

ʂ P value in test for interaction with diabetes in pregnancy in relation to the risk of stillbirth.

**Table 4:** Neonatal mortality in diabetic versus non-diabetic twin pregnancies, U.S. matched multiple birth data 1995-2000 [16].

	**Neonatal mortality (rate**)			
	Diabetic	Non-diabetic	Crude	Adjusted*
	Pregnancy	Pregnancy	HR (95% CI)	HR (95% CI)
	n/total (%)	n/total (%)		
All live births (n=555182)	246/19508 (1.26)	11798/535674 (2.2)	**0.56 (0.49-0.65)**	**0.65 (0.56-0.74)**
Mode of delivery (^ʂ^ P=0.004)				
Caesarean section	87/7544 (1.15)	2796/184966 (1.51)	**0.78 (0.62-0.97)**	0.85 (0.67-1.06)
Vaginal	62/4472 (1.39)	4981/156956 (3.17)	**0.45 (0.34-0.58)**	**0.49 (0.38-0.74)**
Unknown	97/7492 (1.29)	4021/193752 (2.08)	**0.58 (0.46-0.72)**	**0.63 (0.51-0.79)**
Gestational age^ʃ^ (^ʂ^ P<0.0001)				
Very preterm (<32 wks)	178/19508 (0.91)	10222/535674 (1.91)	**0.46 (0.40-0.54)**	**0.54 (0.46-0.63)**
Mild preterm (32-36 wks)	48/17532 (0.27)	1069/474327 (0.23)	1.22 (0.90, 1.67)	1.25 (0.92, 1.70)
Term (>=37 wks)	20/7575 (0.26)	507/237008 (0.21)	1.31 (0.82-2.10)	1.33 (0.83-2.14)
Birth weight (^ʂ^ P<0.0001)				
Very low (<1500 g)	183/1680 (10.89)	10292/54028 (19.05)	**0.51 (0.44-0.60)**	**0.57 (0.49-0.67)**
Low (1500-2499 g)	39/8499 (0.46)	910/236100 (0.39)	1.19 (0.83-1.69)	1.16 (0.81-1.66)
Normal (>=2500 g)	19/9303 (0.20)	332/244533 (0.14)	**1.83 (1.14-2.94)**	**1.81 (1.12-2.93)**
Fetal growth (^ʂ^ P<0.0001)				
SGA (<10^th^)	69/1872 (3.69)	2981/56216 (5.30)	**0.68 (0.53-0.88)**	**0.75 (0.58-0.98)**
AGA (10^th^-90^th^)	165/15047 (1.10)	8485/429510 (1.98)	**0.55 (0.47-0.65)**	**0.63 (0.53-0.74)**
LGA (>90^th^)	12/2589 (0.46)	332/49948 (0.66)	0.65 (0.35-1.22)	0.72 (0.39-1.35)

HR = Hazard ratio; CI = confidence interval

* Hazard ratios adjusted for maternal race, age, education, marital status, parity, smoking, other maternal major illnesses, fetal sex, mode of delivery and twin-cluster level dependence in Cox proportional hazard models.

ʃ Gestational age group-specific mortality rates and hazard ratios were calculated using the fetuses-at-risk denominator including all fetuses at risk of death (both born and unborn babies).

ʂ P value in test for interaction with diabetes in pregnancy in relation to the risk of neonatal mortality.

## Discussion

### Main findings

This is the first report showing that diabetes was associated with an overall reduced risk of perinatal death in twin pregnancies due to a possible survival benefit in very preterm and very low birth weight births, even though there was a significantly increased risk of perinatal death in normal birth weight twins.

### Data interpretation and comparisons with previous findings

It has been reported that unlike singleton pregnancies, diabetes may have some “beneficial” impact on some neonatal outcomes in multiple pregnancies such as a reduced risk of neonatal death [15]. The current study has overcome a major limitation in the previous study (on the risk of neonatal death in live births) which could not address the potential role of “risk shift” - the reduction in neonatal deaths in diabetic pregnancies may be due to a corresponding increase in fetal deaths. We observed an overall reduced risk of both stillbirth and neonatal death in twins comparing diabetic vs. non-diabetic pregnancies, largely due to lower mortality rates in very preterm and very low birth weight high-risk births.

It is somewhat paradoxical that diabetes was associated an overall reduced risk of perinatal death in twin pregnancies, despite higher rates of birth defects and preterm delivery. The mechanisms are unclear. Higher maternal glucose levels may be general beneficial to fetal growth and development in multiple pregnancies due to the greater fuel/nutrient demand, as evidenced by the observed lower risk of SGA birth in diabetic pregnancies especially in those ending in extreme preterm delivery. However, the reduced risk of perinatal death in diabetic pregnancies was more apparent among AGA twins, indicating that better fetal growth may not be an important contributor. Alternatively, there might be more frequent other unmeasured severe maternal illnesses in the non-diabetic group that may account for the elevated risk of perinatal death, but this appears to be highly unlikely as diabetic mothers in general are more prone to have other medical complications. Another possibility is that the hyperglycemia-stimulated fetal endocrine system may be sensitized with stronger capacity for survival when the fetus was delivered in very “immature” conditions. Experimental studies in animal models may help to elucidate the gestational age-specific roles of maternal hyperglycemia on fetal development and survival.

With advancing gestation and the fetus grown larger, the risk pattern reversed. An elevated risk of perinatal death was observed in twin diabetic pregnancies ending in mild preterm or normal weight births. It appears that when the fetus has grown mature enough, continuing living in a diabetic intrauterine environment becomes detrimental, while in early gestation, mild hyperglycemia (most diabetic patients might have received appropriate treatment) may confer some survival “benefit” in multiple pregnancies.

It is unclear why the “protective” effect of diabetes against perinatal death was only observed in vaginal deliveries. We speculate that poorly controlled diabetic women may have higher rates of other complications of pregnancy, such as obesity or fetal macrosomia, and hence may be more likely to undergo cesarean delivery. It is possible that the protective effect of diabetes may be related to the degree of glycemic control or the severity of disease. The “protective” effect may be restricted to women with well-controlled or mild diabetes only.

### Limitations

The U.S. birth data contained no information on clinical subtypes of diabetes in pregnancy (class A1, A2, B or higher), duration of diabetes, type of treatment or the degree of glycemic control. Underreportings of diabetes in pregnancy could be relatively substantial in such large population-based data [20]. However, such underreportings (missed cases) would have created noise variations which would only tend to have blunted the observed risk differences between diabetic and non-diabetic pregnancies. We had no data on clinical management of diabetes. Hyperglycemia, hypoglycaemia and variability in clinical management protocols may affect perinatal outcomes [21-23]. However, it remains uncertain which treatment modality is associated with optimal perinatal outcomes [24,25], and data specific to multiple pregnancies remain absence. Further studies are needed to clarify the effects of glycemic control on perinatal outcomes in diabetic pregnancy.

### Conclusions

Diabetes in pregnancy appears to be “protective” against perinatal death in very premature or very low birth weight twin births. Prospective studies are needed to either clarify the clinical factors associated with this effect and to differentiate the effects of pre-gestational and gestational diabetes, or to determine whether this effect is an artifact of unmeasured confounders.
